# Leucine-Rich Repeat Kinase 2 Influences Fate Decision of Human Monocytes Differentiated from Induced Pluripotent Stem Cells

**DOI:** 10.1371/journal.pone.0165949

**Published:** 2016-11-03

**Authors:** Anna Speidel, Sandra Felk, Peter Reinhardt, Jared Sterneckert, Frank Gillardon

**Affiliations:** 1 CNS Diseases Research, Boehringer Ingelheim Pharma GmbH & Co. KG, Biberach an der Riss, Germany; 2 CRTD / DFG-Center for Regenerative Therapies Dresden, Dresden, Germany; Universita degli Studi di Padova, ITALY

## Abstract

Mutations in *Leucine-rich repeat kinase 2* (*LRRK2*) are strongly associated with familial Parkinson’s disease (PD). High expression levels in immune cells suggest a role of LRRK2 in regulating the immune system. In this study, we investigated the effect of the *LRRK2* (G2019S) mutation in monocytes, using a human stem cell-derived model expressing *LRRK2* at endogenous levels. We discovered alterations in the differentiation pattern of *LRRK2* mutant, compared to non-mutant isogenic controls, leading to accelerated monocyte production and a reduction in the non-classical CD14+CD16+ monocyte subpopulation in the *LRRK2* mutant cells. LPS-treatment of the iPSC-derived monocytes significantly increased the release of pro-inflammatory cytokines, demonstrating a functional response without revealing any significant differences between the genotypes. Assessment of the migrational capacity of the differentiated monocytes revealed moderate deficits in *LRRK2* mutant cells, compared to their respective controls. Our findings indicate a pivotal role of LRRK2 in hematopoietic fate decision, endorsing the involvement of the immune system in the development of PD.

## Introduction

Mutations in *leucine-rich repeat kinase 2* (*LRRK2*) are strongly associated with Parkinson’s disease (PD), the second most prevalent neurodegenerative disorder [1–4]. LRRK2 is a 286 kDa, multidomain and multifunctional protein [5]. Its most frequent mutation, G2019S, accounts for 1% of sporadic and 5% of familial PD cases in Caucasians. The mutation is located in the kinase domain and increases kinase activity of LRRK2 [5–9]. However, the pathomechanisms linking LRRK2 (G2019S) function to Parkinson’s disease are not yet fully understood.

One hallmark of PD is neuroinflammation. Consequently, various studies have investigated a possible link between *LRRK2* and inflammation [10–12]. Upregulation of *LRRK2* in response to pathogenic stimuli [13–17] and increased pro-inflammatory activity has been observed in primary *LRRK2* mutant immune cells [13,18,19]. *LRRK2* knockdown and pharmacological inhibition of LRRK2 alleviated these enhanced inflammatory responses [15,16,20], indicating a pivotal role of the kinase in the immune response.

Within the innate immune response, circulating blood monocytes play an important role. Upon activation, monocytes release a variety of effector molecules, amongst them cytokines and chemokines, to fight pathogenic insults [21]. In the human body three functional subsets of monocytes are known, defined by their expression of CD14 and CD16 (CD14++CD16-, CD14++CD16+ and CD14+CD16+) [22–24]. Recent studies have reported alterations in the distribution of the so-called classical CD14+CD16- and non-classical CD14+CD16+ monocyte subpopulations in peripheral blood samples of PD patients [25,26]. High LRRK2 protein levels, in the CD14+CD16+, compared to the CD14+CD16-, monocyte subpopulation isolated from healthy donors, led to the suggestion of LRRK2 playing a role in activation/maturation of peripheral blood cells [27].

In this study, we differentiated human induced pluripotent stem cells (iPSCs) into monocytes to further investigate perturbations in the immune system associated with mutant *LRRK2*, which might contribute to the development of PD. Using isogenic cell lines, we established a cellular model, displaying the same genetic and epigenetic background in both *LRRK2* mutant and control lines, allowing for direct comparison of gene mutation effects. Additionally, mimicking monocyte development in the dish, the model allowed for studying early phenotypic changes and associated pathological mechanisms, helping to shed light on disease initiation and progression.

## Materials and Methods

### Induced pluripotent stem cells

The *LRRK2* (G2019S) patient-derived iPS cells line, the zinc finger nuclease-mediated gene-corrected isogenic control iPSC line, the non-mutant control iPSC line, and the *LRRK2* (G2019S) knock-in isogenic iPSC line were generated and extensively characterized previously [28]. Informed consent was obtained from all patients prior to cell donation. The Ethics Committee of the Medical Faculty and the University Hospital Tuebingen previously approved this consent form. Karyotypical integrity of the reprogrammed cell lines was validated using an Illumina HumanCytoSNP-12v2 array and the results have been deposited in Gene Expression Omnibus (GEO) under accession number: GSE87462. The analyzed cell lines did not show signs of significant abnormalities.

### iPSC culture and differentiation into monocytes

All cell lines were cultured at 37°C and 5% CO_2_. The cells were maintained in mTeSR-1 (Stem Cell Technologies, Köln, Germany) on hESC-qualified Matrigel-coated dishes (BD Biosciences, Heidelberg, Germany). Passaging was performed upon confluency using 0.02% EDTA (Sigma, Munich, Germany) and cell clumps were replated at a dilution of 1:3 to 1:6.

Differentiation of iPSCs was performed based on a previously published protocol [29]. In brief, embryoid bodies (EBs) were formed in AggreWell^TM^800 plates (Stemcell Technologies) for 4 days with daily changes of mTeSR-1 supplemented with 10 μM Y-27632 (Tocris, Bristol, UK), 50 ng/ml BMP4 (Peprotech, Hamburg, Germany), 20 ng/ml SCF (MACS Milteny Biotech, Bergisch Gladbach, Germany) and 50 ng/ml VEGF (Peprotech). For differentiation into monocytes, EBs were collected in X-VIVO 15 medium (Lonza, Basel, Switzerland), containing 1% GlutaMax (Life Technologies, Darmstadt, Germany), 50 μM 2-Mercaptoethanol (Life Technologies), 100 ng/ml M-CSF (Life Technologies), 25 ng/ml IL-3 (R&D Systems, Abingdon, UK) and 1% Antibiotic-Antimycotic (Life Technologies) and transferred to tissue culture treated 6-well plates (Thermo Scientific, Darmstadt, Germany). Three 6-well plates of each cell line, containing 10–12 EBs per well, were used for differentiation. A 50% medium change was performed every 5–7 days. Monocytes were harvested weekly from the supernatant.

### qRT-PCR

iPSC-derived monocytes were lyzed in RLT buffer (Qiagen, Hilden, Germany) containing 1% β-mercaptoethanol (Roth, Karlsruhe, Germany). RNA was isolated using the RNeasy mini kit in combination with QIAshredder columns and the RNase-free DNAse set (all Qiagen) according to the manufacturer’s protocol. RNA concentration was measured using a NanoDrop 1000 spectrophotometer (Thermo Scientific, Wilmington, USA). Each cDNA synthesis was performed with 1.4 μg RNA sample, using the SuperScript VILO cDNA synthesis kit (Thermo Fisher Scientific, Schwerte, Germany) according to the manufacturer’s protocol. cDNA synthesis was verified by measuring the concentration using the NanoDrop 1000 spectrophotometer. RT-PCR was then performed in triplicates, using 40 ng cDNA per reaction, the TaqMan QuantiFast Probe PCR Kit (Qiagen) and the human *LRRK2* Assay-On-Demand (Hs00968209_m1) as well as the human *RNA polymerase II* Assay-On-Demand (Hs01558819_m1; both Thermo Fisher Scientific). The following cycling conditions were used: 2 min at 50°C, 2 min at 95°C, 15 sec at 95°C and 1 min at 60°C with 40 repeats of the last two steps (7900HT Sequence detection system, ABI Prism, Foster City, USA). *LRRK2* expression levels relative to *RNA polymerase II* expression were determined using the 2(-Delta Delta C(T)) Method [30].

### Gel electrophoresis and immunoblotting

iPSC-derived monocytes were lyzed in RIPA-2 buffer (Alfa Aesar, Karlsruhe, Germany) supplemented with Protease Inhibitor Cocktail and Phosphatase Inhibitor Cocktail 2 (both 1:100, Sigma). Proteins were resolved by electrophoresis on 4 to 12% NuPAGE Bis-Tris gradient gels according to the manufacturer’s protocol, using NuPAGE MOPS running buffer (Life Technologies). The proteins were blotted onto nitrocellulose membranes (Life Technologies), followed by incubation in blocking buffer (5% skimmed milk powder in Tris-buffered saline containing 0.1% Tween-20) for 1 hour at room temperature. The membranes were then incubated with antibodies against LRRK2 (Rb mAB MJFF2 (c41-2), #ab133474, Abcam, Cambridge, UK), pLRRK2(Ser935) (Rb mAB UDD 10(12)J(phosphoS935), #ab133450, Abcam), pLRRK2(S1292) (Rb mAB MJFR-19-7-8, #ab203181, Abcam) or β-Actin (clone AC-74, #A5316, Sigma) overnight at 4°C. Horseradish peroxidase-conjugated secondary antibody and enhanced chemiluminescence reagents were used for detection (Western Lightning Plus-ECL Kit, Perkin Elmer, Walluf, Germany). Protein transfer and comparable protein load was verified using a protein staining kit (MemCode, Thermo Scientific). Densitometric analysis of the immunoblots was performed using Quantity One software (Biorad, Munich, Germany).

### Leukocyte differential analysis

iPSC-derived monocytes were collected and analyzed in triplicates within their conditioned medium using an Advia120 Hematology system (Siemens Healthcare, Erlangen, Germany), a flow-cytometry-based hematology instrument using two distinct methods to analyze whole blood samples. In the peroxidase channel, peroxidase-positive cells (neutrophils, eosinophils and monocytes) were distinguished from peroxidase-negative cells (lymphocytes and basophils). In the peroxidase cytogram, cell size was plotted against peroxidase activity. In the basophil channel, cells were stripped and classified according to size and nuclear density which was then plotted against each other in the basophil cytogram. Proportions of detected cell types were calculated by means of a probe-specific calibration factor followed by cluster analysis. Gates were set based on tentative calculations executed by the software.

### Flow Cytometry

Flow cytometric analysis was performed based on manufacturer’s instructions and previously published protocols (http://static.bdbiosciences.com/documents/BD_

Protocol_CellSurface_Staining_StemCell.pdf?_ga = 1.233729431.884400444.1467102281; http://www.bdbiosciences.com/us/resources/s/cellsurface; [31,32]). In brief, iPSC-derived monocytes were harvested, resuspended in PBS supplemented with 1.5% ES-qualified FCS to block unspecific binding and stained for 30 min at 4°C with specific antibodies. In addition to unstained controls, the respective isotype-matched antibodies were used to control for nonspecific binding [32]. Cells were washed, resuspended in PBS with ES-FCS and phenotypically analyzed on a LSR II flow cytometer (Beckton Dickinson, Heidelberg, Germany). The following monoclonal anti-human antibodies were used: CD14-APC, CD14-FITC, CD16-FITC and CD45-PerCP-Cy5.5 (BD Pharmingen, Heidelberg, Germany). Gates determining the monocyte population were defined within the CD45+ and CD14+ scatter plot, respectively. The positive cell populations were then identified in the forward (FSC) versus side (SSC) scatter plot and confirmed by back-gating CD14+CD45+ events into the scatter plot. Applying this strategy, a monocyte gate based on morphology (size, granularity) could be determined, excluding autofluorescent dead cells and cell debris. A minimum of 5 x 10^4^ gated events were acquired per sample. Within this population, percentages of cells, which stained positive for the respective markers, as well as mean fluorescent intensities were determined. For sorting experiments, harvested monocytes were stained with FITC-conjugated anti-human CD14 antibody (BD Pharmingen) as described above. Immunolabeled cells were sorted on a FACS Aria II instrument (Becton Dickinson) within a monocyte gate defined in the scatter plot. A minimum of 1 x 10^4^ events were recorded in parallel for analysis of mean fluorescence intensities and percentages of the monocyte population.

Data analysis was performed using FACSDiva Software (Becton Dickinson) and Flowing Software v2.5.1 (University of Turku, Finland).

### Gene expression analysis

Gene expression analysis of FACS-sorted, CD14+ iPSC-derived monocytes (16–19 weeks of differentiation) was performed as published previously [33]. In brief, cells were lyzed in RLT buffer containing 1% β-mercaptoethanol and RNA was isolated in replicates of four, using the RNeasy Micro Kit (Qiagen) according to the manufacturer’s instructions. Preparation of the mRNA sequencing library was conducted using the TrueSeq RNA Sample Preparation Kit v2 (RS-122-2002, Illumina Inc, San Diego, USA) and sequencing was performed using the TruSeq SBS Kit HS e v3 (FC-401-3002, Illumina Inc.) on an Illumina HiSeq2000 instrument. The criteria used to identify genes which were expressed in iPSC-derived monocytes were mean reads per kilo base per million (RPKM) > 5.

### Cytokine release assay

FACS-sorted, CD14+ iPSC-derived monocytes were plated in triplicates in 96-well plates at a density of 2.5 x 10^4^ cells per well. At day 7, the cells were treated with 100 ng/ml LPS (Sigma) as previously described [13,34,35], a concentration which has been shown to increase LRRK2 protein levels [15]. After 6 hours, a time point of substantially increased release of the relevant cytokines [15,34,35] but before maximal release [36–38], cell culture supernatants were collected, avoiding a ceiling effect. IFNγ, IL-1β, IL-6 and TNFα levels in biological triplicates of treated samples and controls were quantified using an electrochemiluminescence immunoassay (Meso Scale Discovery, Gaithersburg, USA) as previously described [13].

### Transwell migration assay

FACS-sorted, CD14+ iPSC-derived monocytes were plated in triplicates in the upper wells of a 96-well Transwell plate with a pore size of 8.0 μm (Corning, Munich, Germany) at a density of 2.5 x 10^4^ cells per well. The cells were allowed to rest for 30 min. Titration of both ADP and ATP concentrations identified 100 μM ATP as reliable stimulus causing migration of iPSC-derived monocytes when supplemented into the monocyte maintenance medium in the bottom wells. Based on the Transwell Cell Migration, Chemotaxis and Invasion Assay Protocol (Corning; http://csmedia2.corning.com/LifeSciences/media/pdf/protocol_CLS_AN_211_CellMigration_Chemotaxis_InvasionAssay_Using_Staining.pdf), the cells were allowed to migrate for 24 hours. Thereafter, cells, which have not migrated were removed from the inserts and migrated cells, which were still attached to the bottom side of the membrane were dissociated into the medium of the lower well. All migrated cells in the lower wells were stained with Calcein AM (Life Technologies). After incubation for 30 min at RT in the dark, the Calcein signal of all migrated cells was measured using an EnVision Multilabel Plate Reader (Perkin Elmer). The migration factor was calculated in relation to the mean migration of non-mutant control cells without the chemotactic stimulus ATP.

### Statistical analysis

Statistical analysis was performed using Graph Pad Prism software version 6.01 for Windows. All values are presented as mean ± SEM unless stated otherwise. Statistical significance was determined using the appropriate two-tailed *t*-test or analysis of variance (ANOVA), respectively. Statistical significance was set at p < 0.05.

## Results

### *LRRK2* (G2019S) accelerates differentiation of human iPS cells towards monocytes

Interested in LRRK2-mediated immune pathomechanisms, we differentiated in parallel iPS cells reprogrammed from PD patient fibroblasts containing the *LRRK2* (G2019S) mutation, iPS cells containing a ZFN-mediated *LRRK2* (G2019S) knockin, and the corresponding isogenic control cell lines, into monocytes. The differentiated cells were collected from the supernatant of the differentiation cultures and their cellular identity was analyzed using flow cytometry.

**Fig 1:**
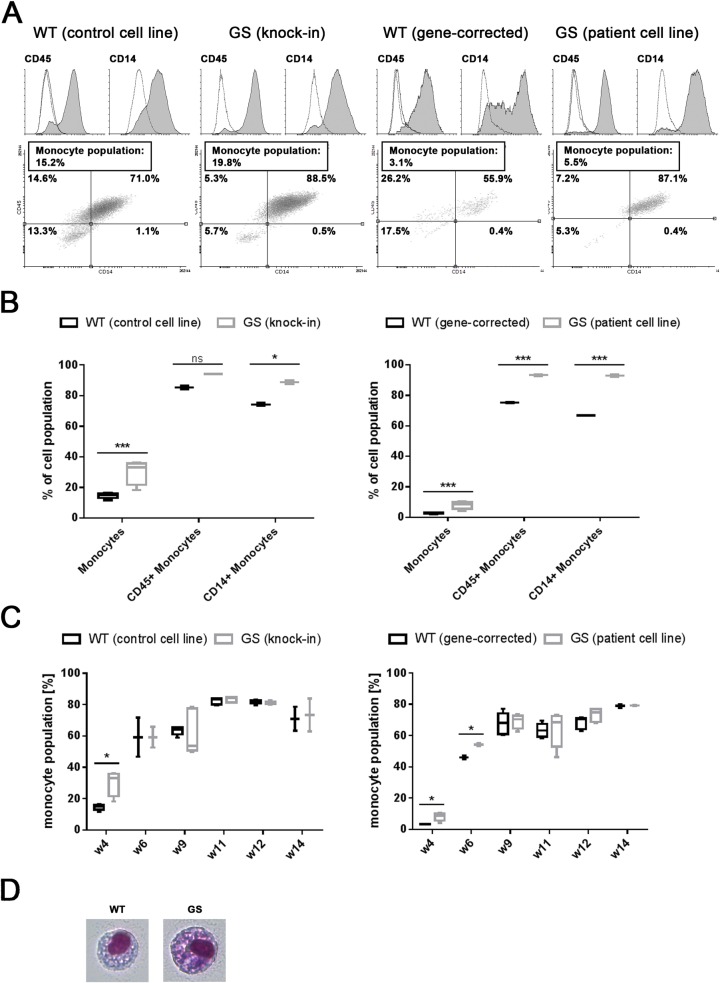
Accelerated differentiation towards the monocytic lineage in *LRRK2* (G2019S) iPS cells compared to non-mutant controls. (A) Representative peaks of flow cytometry analysis of the hematopoietic lineage marker CD45 and the monocyte marker CD14 reveal monocyte identity of the iPSC-derived cells after 4 weeks of differentiation in both *LRRK2* (G2019S) mutant (GS) and non-mutant control (WT) cell lines. Histograms represent specific surface marker staining (shaded grey) compared to unstained (dashed line) and isotype-matched (solid line) controls. Representative CD45 vs. CD14 scatter plots illustrate the distribution of the gated monocyte population. The respective monocyte yields (differentiation efficiency) are given in boxes. (B) After 4 weeks of differentiation, more *LRRK2* (G2019S) mutant cells (GS; left panel: 30.2%, right panel: 8.0%) differentiated into monocytes compared to non-mutant controls (WT; left panel: 14.7%, right panel: 3.0%). The percentage of CD45+ and CD14+ cells within the monocyte population is significantly higher in *LRRK2* (G2019S) patient (GS) cells compared to the gene-corrected control (WT) (CD45: p < 0.001, CD14: p < 0.001; right panel). Similarly, comparison of the LRRK2 (G2019S) knock-in (GS) and the control cell line (WT) revealed increased CD45 and CD14 expression (CD45: p > 0.05, CD14: p < 0,05) (C) Analysis of the efficiency of monocyte differentiation over time revealed significant differences during early differentiation in both groups followed by comparable monocyte yields starting from week 6–9. (D) Representative Pappenheim stainings of iPSC-derived monocytes show no morphological difference between *LRRK2* (G2019S) (GS; right panel) and non-mutant cells (WT; left panel). Pooled data of 2–5 independent experiments is shown in box plots (min to max), error bars represent mean ±SEM, *p<0.05, **p> 0.01, ***p<0.001. For each cell line, three 6-well plates, with each well containing 10–12 monocyte producing cell clusters, were set up for differentiation and analyzed in independent experiments.

After four weeks of differentiation, cells were harvested from the differentiation cultures for the first time. Surface expression of the hematopoietic lineage marker CD45 and the monocyte marker CD14 [22,39] confirmed successful differentiation of the iPS cells (Fig 1A and 1B). The monocyte population was determined based on the cellular morphology of the recorded iPSC-derived cells, according to their distribution in the scatter plot confirmed by subsequent back-gating (S1A Fig). The average monocyte proportion in the recorded *LRRK2* (G2019S) mutant cells was twice as high as in the respective isogenic control cells (‘monocytes’; LRRK2 patient cell line: 8.0% vs. gene-corrected control: 3.0% and LRRK2 (G2019S) knock-in: 30.2% vs. control cell line: 14.7%; Fig 1B). Within the monocyte population, significantly more (p < 0.001) *LRRK2* mutant patient cells expressed the surface marker CD45, compared to the gene-corrected isogenic control (93.3% vs. 75.2%; Fig 1B, right panel). Similarly, the *LRRK2* (G2019S) knock-in cells expressed more CD45 than the healthy control cell line (88.8% vs. 74.2%; p < 0.05; Fig 1B, left panel). The difference in CD45 mean fluorescent intensities reached significance in all cell lines (p < 0.01 and p < 0.001, respectively; S1B Fig). Furthermore, the pronounced differences in both percentages of CD14+ cells (LRRK2 patient cell line: 93.0% vs. gene-corrected control: 66.8% and LRRK2 (G2019S) knock-in: 88.8% vs. control cell line: 74.2%; p < 0.05 and. p < 0.001, respectively; Fig 1B) and CD14 mean fluorescent intensities (p < 0.01 and. p < 0.001, respectively; S1B Fig) suggested differences in the differentiation pattern between both genotypes at early time points.

FACS analysis revealed that the iPSC-derived cellular population harvested after four weeks of differentiation represented a heterogeneous population consisting of cells of different sizes and morphologies. Concluding that the protocol did not generate iPSC-derived monocytes exclusively, we asked whether the harvested cell population mainly consisted of immature precursor cells rather than cells displaying mature monocyte identity. To answer this question, the differentiation cultures were assessed over time and iPSC-derived cell populations were harvested at different time points.

FACS analysis of cell samples harvested after six weeks of differentiation time indicated that the cultures increased their efficiency in producing monocytes with longer differentiation time. Fig 1C shows the higher proportion of monocytes in *LRRK2* mutant lines at early differentiation time points, whereas in all lines monocyte production increased to a similar plateau after about 9 weeks and the differentiation cultures reliably generated about 80% monocytes over a time period of 8–10 weeks.

Pappenheim staining exhibited comparable morphologies with a high cytoplasm-to-nucleus ratio in both genotypes, confirming published data from iPSC-derived monocytes [29,40] (Fig 1D). In addition to characterization based on cellular morphology and surface marker expression, myeloid cells (including stem cell-derived myeloid cells) are classified using peroxidase staining [31,41]. Using an Advia120 Hematology system, leukocyte differential analysis was performed on harvested cells, confirming both monocyte identity and an accelerated differentiation pattern of *LRRK2* mutant cells (S2A and S2B Fig). Due to the fact that iPSC-derived monocytes may differ from natural peripheral blood leukocytes, which may lead to categorization problems performing automated leukocyte differential analysis, we additionally investigated gene expression of the differentiated monocytes focussing on genes which are known to identify monocytes, but also neutrophils, T and B cells. In addition to CD45 and CD14, which have been investigated in the FACS analysis experiments, specific monocyte markers, like CD163, a receptor involved in clearance of haemoglobin and regulation of cytokine production [42], the macrophage colony-stimulating factor receptor (CSFR1), the transmembrane receptor CD33, the toll-like receptor 4 (TLR4) and the low affinity IgG Fc region marker CD16 were expressed in the iPSC-derived monocytes after 16–19 weeks of differentiation (S2C Fig). Furthermore, the HLA class II histocompatibility antigen subunit genes HLA-DRA, HLA-DRB1 and HLA-DRB6 were detected. Further, CD4 mRNA, expressed by monocytes, granulocytes and T cells was detected whereas the T cell markers CD3 and CD8 were not expressed. Genes characterizing B cells (CD19, CD20) or neutrophils (CD16b, CD177, CD203c) were not expressed (RPKM < 5; markers reviewed by the HLDA workshops (www.hcdm.org) and described in www.bdbiosciences.com/documents/cd_marker_handbook), indicating monocytes being the prevalent population after 16–19 weeks.

Taken together, we could observe small, but consistent phenotypic differences during the differentiation of iPS cells, with *LRRK2* (G2019S) mutant cells differentiating faster towards the monocyte lineage during the early differentiation phase, compared to their respective isogenic controls.

### Monocyte subpopulations differ between *LRRK2* (G2019S) mutants and controls

**Fig 2:**
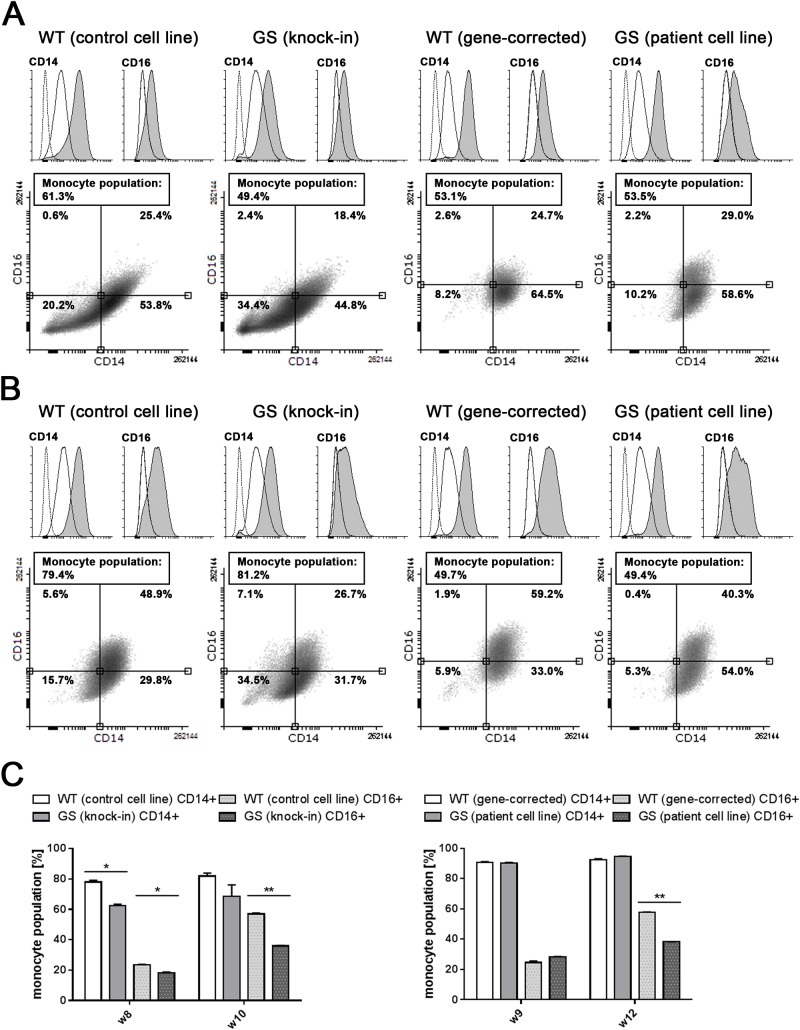
Differences in monocyte subtype ratios between iPSC-derived monocytes. Representative peaks of flow cytometric analysis of the LPS receptor CD14 and the low affinity IgG Fc region marker CD16 in both non-mutant control (WT) and isogenic *LRRK2* (G2019S) mutant (GS) cells after 8–9 weeks (A) and 10–12 weeks (B) of differentiation. Histograms represent specific surface staining (shaded grey) compared to unstained (dashed line) and isotype-matched (solid line) controls. Representative CD16 vs. CD14 scatter plots illustrate the distribution of the gated monocyte population. The respective monocyte yields (differentiation efficiency) are given in boxes. (C) Percentage of CD14+ and CD16+ cells within the monocyte population of non-mutant control (WT) versus *LRRK2* (G2019S) mutant (GS) cells analyzed in week 8 and 10 (left panel) and 9 and 12 (right panel) of differentiation, respectively. CD16 surface expression is significantly higher (p < 0.01) in non-mutant (WT) cells in week 10 and 12, compared to *LRRK2* (G2019S) mutant (GS) cells. Error bars represent mean +SEM; *p>0.05, **p<0.01. For each cell line, three 6-well plates, with each well containing 10–12 monocyte producing cell clusters, were set up for differentiation and analyzed in two independent experiments.

In the human body, monocytes differentiate into various subtypes, which can be distinguished according to their surface receptor expression [22]. The CD14+CD16+ non-classical monocytes subpopulation is reduced in peripheral blood samples of PD patients, whereas CD14+CD16- classical monocytes are enriched compared to healthy controls [25]. Thus, we sought to characterize these subpopulations within our iPSC-derived monocytes. Flow cytometry analysis of the LPS co-receptor CD14 and the low affinity IgG Fc region marker CD16 after 9 weeks of differentiation, revealed no differences in expression levels of CD14 or CD16 in the *LRRK2* (G2019S) patient (CD14: 90.7%, CD16: 24.5%) versus the isogenic gene-corrected cell line (CD14: 90.4%, CD16: 28.4%; p > 0.05; Fig 2A, WT (gene-corrected) and GS (patient cell line) and Fig 2C, right panel). After 12 weeks, when monocyte yields were similar in differentiation cultures of the respective *LRRK2* mutant and non-isogenic control cell lines (Fig 1C), *LRRK2* (G2019S) patient monocytes showed significantly lower (p < 0.01) expression of CD16 (38.25%) compared to the gene-corrected controls (57.7%) (Fig 2B, WT (gene-corrected) and GS (patient cell line) and Fig 2C, right panel). Having observed that the *LRRK2* (G2019S) knock-in and the respective isogenic control cell line differentiate faster towards monocytes (Fig 1C), monocytes derived from these lines were analyzed at earlier time points. After 8 weeks of differentiation, 23.7% of non-mutant control monocytes expressed CD16, whereas *LRRK2* (G2019S) knock-in cells expressed significantly less CD16 (18.25%; Fig 2A, WT (control cell line) and GS (knock-in) and Fig 2C, left panel). Analysis of the cells after 10 weeks of differentiation revealed significantly lower (p < 0.01) CD16 expression in *LRRK2* (G2019S) knock-in monocytes (36.0%) compared to non-mutant control cells (57.0%), similar to what has been observed in the other two cell lines (Fig 2B and 2C). Due to our gating strategy, that enabled us to only analyze the monocyte population, and due to the fact that all CD16+ cells were CD14+, CD16 expression could be attributed solely to CD14+CD16+ monocytes. Thus, our findings are consistent with the abovementioned data from blood samples of sporadic PD patients [25]. Harvested cells were analyzed also at later time points (S3 Fig), however, at that time, the monocyte yield from our differentiation cultures decreased and the monocyte producing cell clusters showed morphological signs of degeneration, precluding meaningful analysis.

### iPSC-derived monocytes display similar *LRRK2* levels in LRRK2 (G2019S) mutants and controls

**Fig 3:**
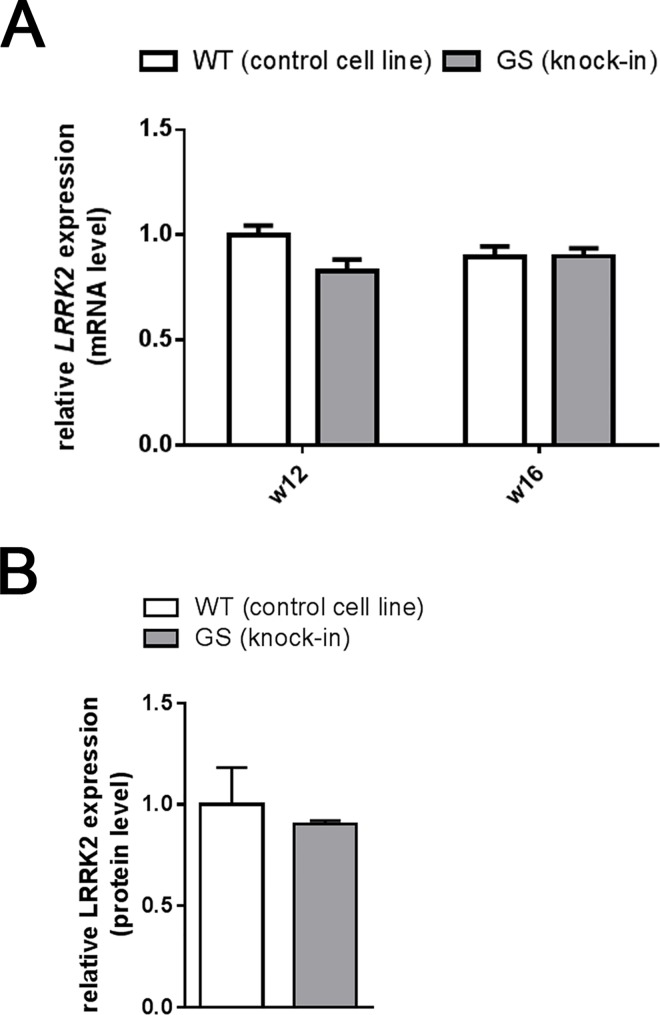
LRRK2 and phospho-LRRK2 levels in iPSC-derived monocytes. (A) *LRRK2* mRNA expression relative to RNA polymerase II expression was determined using the 2(-Delta Delta C(T)) method. The relative expression is shown for non-mutant control (WT) and LRRK2 (G2019S) knock-in (GS) cells (n = 3). Data was normalized to the non-mutant control in week 12. No differences between the isogenic cell lines were detected (p > 0.05). (B) Densitometric analysis of immunoblots of three independent cultures per genotype reveal similar LRRK2 protein expression levels (p > 0.05). Error bars represent mean +SEM, *p<0.05.

Using isogenic cell lines in this study, we precluded genetic and epigenetic differences to account for the observed phenotype during the differentiation of iPS cells towards monocytes. To ensure that the diverging differentiation pattern was related to the G2019S point mutation in the *LRRK2* gene, the expression pattern in iPSC-derived monocytes was examined at various time points. Real-time quantitative PCR (qRT-PCR) revealed similar expression levels of *LRRK2* mRNA in all cell lines used in this study, a representative example is shown in Fig 3A. Western Blotting additionally confirmed LRRK2 expression on protein level. No significant differences in total protein expression were found between both genotypes following normalization to protein load (Fig 3B).

S935 is not an autophosphorylation site of LRRK2, however, autophosphorylation at S1292 has recently been demonstrated [43,44]. Using commercially available antibodies we detected S1292 phosphorylation in LRRK2 overexpressing cells but not in lysates of iPSC-derived monocytes (S4 Fig).

### Functional analysis of LRRK2 (G2019S) mutant iPSC-derived monocytes

**Fig 4:**
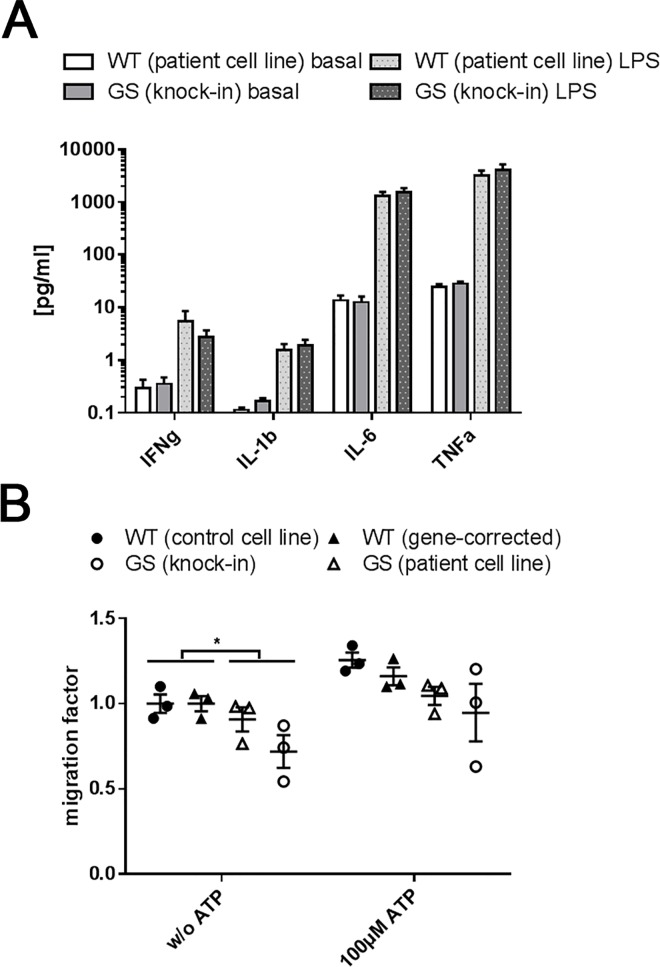
Functional analysis of iPSC-derived monocytes. (A) Release of the pro-inflammatory cytokine IFNγ showed a trend towards an increase after LPS stimulation (100 ng/ml for 6 hours) as measured by ELISA after 11 weeks of differentiation (n = 3). For IL-1β, IL-6 and TNFα the LPS-induced increase reached significance (IL-1β: p < 0.05; IL-6: p < 0.01; TNFα: p < 0.05). No difference was observed upon comparison of LRRK2 (G2019S) mutant (GS) versus non-mutant control (WT) monocytes. (B) *LRRK2* (G2019S) cells (GS) monocytes showed significantly reduced (p < 0.05) basal migration compared to control (WT) after 11–18 weeks of differentiation. The trend towards reduced migration upon exposure to the chemotactic stimulus 100 μM ATP did not reach significance (p < 0.05; n = 3).

Finally, we investigated the functional consequences of the shift between the non-classical versus classical monocyte distribution in *LRRK2* mutant iPSC-derived monocytes compared to their respective non-mutant isogenic controls. In previous studies, we observed increased release of pro-inflammatory cytokines from microglia and leukocytes isolated from LRRK2 mutant mice [13,18]. Additionally, upregulation of LRRK2 activity has been shown to be associated with inflammatory responses in activated peritoneal mouse macrophages [19]. Therefore, we characterized the release of pro-inflammatory cytokines from our iPSC-derived monocytes using an enzyme-linked immunosorbent assay (ELISA) (Fig 4A). In absence of pro-inflammatory stimuli, only low levels of IFNγ, IL-1β, IL-6 and TNFα with no differences between the genotypes were measured. After exposing the cells to the CD14 ligand LPS (100 ng/ml) for 6 hours, a time span after which substantial responses were shown by others [15,34,35] and a ceiling effect was avoided [36–38], the release of IFNγ, IL-1β, IL-6 and TNFα increased (IL-1β: p < 0.05, IL-6: p < 0.01, TNFα. p < 0.05, IFNγ: p > 0.05). Unexpectedly, no difference in the LPS-induced inflammatory response of *LRRK2* mutant (GS) compared to non-mutant (WT) control cells was observed. Our gene expression analysis of TLR4 and MyD88, implicated in LPS activated signaling [45], indicated unaltered mRNA levels comparing the genotypes.

Immune cells are able to recognize foreign structures and injury sites by constantly surveilling their environment [46,47]. Chemotactic agents, like ADP, ATP and UTP, released from damaged cells, induce migration of the patrolling cells [47,48]. To assess the migration capacity of our iPSC-derived monocytes, we tested concentrations ranging between 1–100 μM of ADP and ATP for their suitability as chemotactic stimuli. 100 μM ATP moderately stimulated migration of the iPSC-derived monocytes. Moreover, when combining the data from the two *LRRK2* (G2019S) mutant cell lines and the two control lines, respectively, *LRRK2* mutant monocytes showed a significant (p < 0.05), approximately 20% reduction of migration activity at basal state. This trend did not reach significance upon exposure to 100 μM ATP (Fig 4B).

## Discussion

Our data reveals accelerated differentiation towards monocytes in *LRRK2* (G2019S) mutant iPS cells compared to their non-mutant isogenic controls. The acceleration of fate decision is consistent with prior findings from our lab, indicating a role of LRRK2 in mouse embryonic stem cell differentiation [49]. Furthermore, the involvement of LRRK2 during early monocyte lineage commitment in our in vitro study is consistent with the higher monocyte precursor percentage found in peripheral blood samples of idiopathic and *LRRK2* mutant PD patients [26]. Similar to our cell culture study, these changes in blood monocytes in PD patients were caused by altered differentiation rather than total numbers of mature monocytes. Moreover, in *LRRK2* (G2019S)-derived monocytes analyzed in the present study, a higher percentage of CD14+CD16- cells has been observed, compared to the non-mutant isogenic controls. Similar observations recently have been reported in blood samples from idiopathic PD patients [25]. The influence of LRRK2 activity on the different subtypes of blood monocytes has been characterized in ex vivo studies by Thévenet and colleagues. Using non-specific small molecule LRRK2 inhibitors they linked higher LRRK2 kinase activity to a shift towards the CD14+CD16+ population [27]. This seemingly contradictory finding might be due to the transient effect exerted by LRRK2 during early cell fate decision that has been observed by us and others [26].

CD16+ monocytes are the patrolling monocyte subpopulation in the human blood [50]. Thus, it could be hypothesized that diminished numbers in PD patients might lead to impaired immune surveillance. In addition to less patrolling cells, deficits in migration activity in *LRRK2* (G2019S) mutant mouse microglia, lead to impaired responses to injury [51]. Contradictory findings were reported by Moehle and colleagues [19] showing increased chemotaxis in *LRRK2* (G2019S) mutant peritoneal macrophages. It might be speculated that the influence of LRRK2 on cellular motility may differ dependent on cell type, the microenvironment and the migratory stimulus. Several studies demonstrated that decreased recruitment of immune cells leads to insufficient defense against intruding microbial and viral structures as well as insufficient removal of detrimental, endogenous protein agglomerates and cell debris, ultimately releasing neurotoxic substances into the microenvironment and contributing to neurodegeneration [51–53].

Similar to findings by Moehle et al. in LRRK2 (G2019S) murine peritoneal macrophages [19], we did not observe increased release of pro-inflammatory cytokines in human *LRRK2* (G2019S) mutant iPSC-derived monocyte cultures. By contrast, LRRK2 is associated with activation of immune cells [14,54] and increased inflammatory responses have been found in peripheral immune cells of *LRRK2* mutant mice by us and others [13,15,18]. Several studies linked LRRK2-deficiency or LRRK2 inhibition to decreased cytokine release from activated mouse macrophages [55] and microglia [15,20,56,57], whereas Liu et al. [58] reported increased cytokine release from activated bone marrow-derived macrophages isolated from LRRK2-deficient mice. These studies, however, differed regarding the cell populations and applied stimuli under investigation, indicating that LRRK2 possibly exerts different effects under distinct experimental conditions.

In summary, our findings lead to the hypothesis that mutant LRRK2 may (transiently) affect immune cell maturation and function in an early phase. Interestingly, increased microglial activity in early PD patients was detected in two neuroimaging studies [59,60]. Longitudinal imaging revealed no persistent increase during disease progression [60], indicating that transient alterations in the immune system might contribute to early disease development.

However, it should be kept in mind, that LRRK2 is implicated in other inflammatory disorders and susceptibility to infections (reviewed by [61–63]) and further work is needed to correlate patient data to disease measures and clinical onset of disease. As pointed out previously [28], using isogenic iPS cell lines favored detection of phenotypic differences in the present study which may be masked by the heterogenous genetic background in studies using *LRRK2 (G2019S)* patient samples. It should be noted, though, that the kinetics and yield of myeloid differentiation starting from iPS cells is affected by the genetic background as has been observed in this study and has been reported by others [64]. Performing future patient studies, large sample sizes will be needed to reveal phenotypic differences. A first attempt has been made to compare peripheral blood samples of idiopathic and *LRRK2 (G2019S)* associated PD and healthy controls in a comprehensive transcriptional profiling study [65]. Pathway analysis revealed an upregulation of the complement pathway in blood cells from idiopathic and LRRK2 mutant patients [65]. Interestingly, expression of complement genes (C1QA, C1QB, C1QC) was also upregulated in our LRRK2 mutant iPSC-derived monocytes.

Thus, our human iPSC-derived monocyte model may be suitable to further investigate early phenotypic changes of innate immune cells contributing to PD. These studies will not only contribute to the understanding of the (patho)physiological role of LRRK2, but to PD in general by using iPSC-derived monocytes from sporadic patients.

## Supporting Information

S1 Fig FACS analysis of iPSC-derived monocytes (4 weeks of differentiation time). (A) Illustration of the applied gating strategy to determine the monocyte population. All recorded events were plotted in a CD14-FITC vs. CD45-PerCPCy5.5 dot plot (upper panels). Positive populations were back-gated (illustrated using arrows) to a FSC/SSC dot plot (population ‘Monocytes’, red). This was done for each staining separately (CD45: left column; CD14: middle column). The ‘Monocytes’ population (lower row panels) was selected from the FSC/SSC dot plot (middle row), based on the previous back-gating and exclusion of autofluorescent dead cells and cell debris. This selected population was the same for all samples. The ‘monocyte’ population (without autofluorescent dead cells and cell debris) was displayed in the respective histograms. (B) FACS analysis of the monocyte population after 4 weeks of differentiation time. The mean fluorescent intensity of both CD45 and CD14 is significantly higher in *LRRK2* mutant cells compared to the gene-corrected controls (WT) (CD45: p < 0.0001 and p < 0.01, respectively; CD14: p < 0.001 and p < 0.01, respectively). For each cell line, three 6-well plates, with each well containing 10–12 monocyte producing cell clusters, were set up for differentiation and analyzed in independent experiments
(TIF)

S2 Fig Leukocyte differential analysis of iPSC-derived monocytes using the Advia120 Hematology analytical system. (A) ratios from white blood cell count (WBC) of the differentiated monocytes reveal a higher proportion of monocytes in *LRRK2* (G2019S) (GS) patient cell cultures compared to gene-corrected wild-type (WT) control cultures after 6 weeks of differentiation (left panel; n = 3). After 9 weeks of differentiation, monocytes are the predominant cell type in cultures of both genotypes (right panel). Differences between genotypes are no longer observed. (B) Representative peroxidase (left column) and basophil (right column) cytograms obtained from leukocyte differential analysis of iPSC-derived monocytes. N: Neutrophils; L: Lymphocytes; M: Monocytes; E: Eosinophils; B: Basophils; LUC: large unstained cells; MN: mononuclear cells; PMN: polymorphonuclear cells. (C) Gene expression analysis of CD14+ FACS-sorted iPSC-derived monocytes after 16–19 weeks of differentiation. Genes with mean reads per kilo base per million (RPKM) > 5 were considered being expressed (n = 4; ±SEM).
(TIF)

S3 Fig FACS analysis of iPSC-derived monocytes after 12 weeks of differentiation time. (A) Representative peaks of flow cytometric analysis of CD14 and CD16 in *LRRK2* (G2019S) patient (GS) and gene-corrected control (WT) cells after 19 weeks of differentiation. Histograms represent specific surface staining (shaded grey) compared to unstained (dashed line) and isotype-matched (solid line) controls. Representative CD16 vs. CD14 scatter plots illustrate the distribution of the gated monocyte population, the respective monocyte yields (differentiation efficiency) are given in boxes. For each cell line, three 6-well plates, with each well containing 10–12 monocyte producing cell clusters, were set up for differentiation and analyzed in two experiments.
(TIF)

S4 Fig Phospho-LRRK2(S1292) levels in iPSC-derived monocytes. Representative immunoblots of iPSC-derived monocyte and tagged LRRK2 overexpressing cell lysates showing total LRRK2 (upper lane) and phospho-LRRK2(S1292) (lower lane) protein expression. Similar protein load was verified using Memcode total protein staining. Phospho-LRRK2(S1292) did not reveal any signal in iPSC-derived monocyte lysates.
(TIF)
