# Supplementary figures and images for: Leucine-Rich Repeat Kinase 2 Influences Fate Decision of Human Monocytes Differentiated from Induced Pluripotent Stem Cells
Source: PLoS One. 2016 Nov 3;11(11):e0165949. doi: 10.1371/journal.pone.0165949 (PMC5094768; doi:10.1371/journal.pone.0165949)

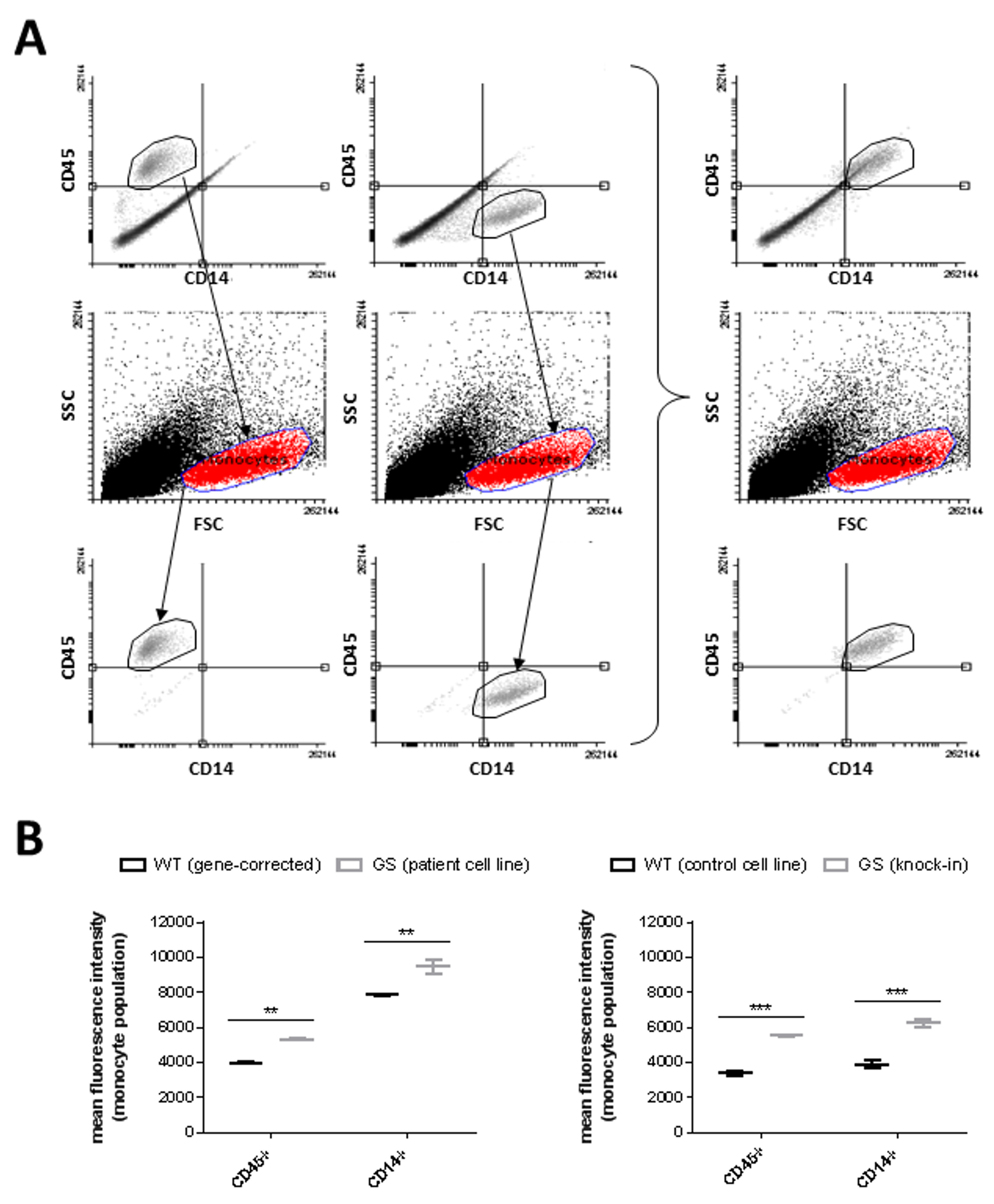

Supplement: S1 Fig — (A) Illustration of the applied gating strategy to determine the monocyte population. All recorded events were plotted in a CD14-FITC vs. CD45-PerCPCy5.5 dot plot (upper panels). Positive populations were back-gated (illustrated using arrows) to a FSC/SSC dot plot (population ‘Monocytes’, red). This was done for each staining separately (CD45: left column; CD14: middle column). The ‘Monocytes’ population (lower row panels) was selected from the FSC/SSC dot plot (middle row), based on the previous back-gating and exclusion of autofluorescent dead cells and cell debris. This selected population was the same for all samples. The ‘monocyte’ population (without autofluorescent dead cells and cell debris) was displayed in the respective histograms. (B) FACS analysis of the monocyte population after 4 weeks of differentiation time. The mean fluorescent intensity of both CD45 and CD14 is significantly higher in LRRK2 mutant cells compared to the gene-corrected controls (WT) (CD45: p < 0.0001 and p < 0.01, respectively; CD14: p < 0.001 and p < 0.01, respectively). For each cell line, three 6-well plates, with each well containing 10–12 monocyte producing cell clusters, were set up for differentiation and analyzed in independent experiments (TIF) [file pone.0165949.s001.tif]

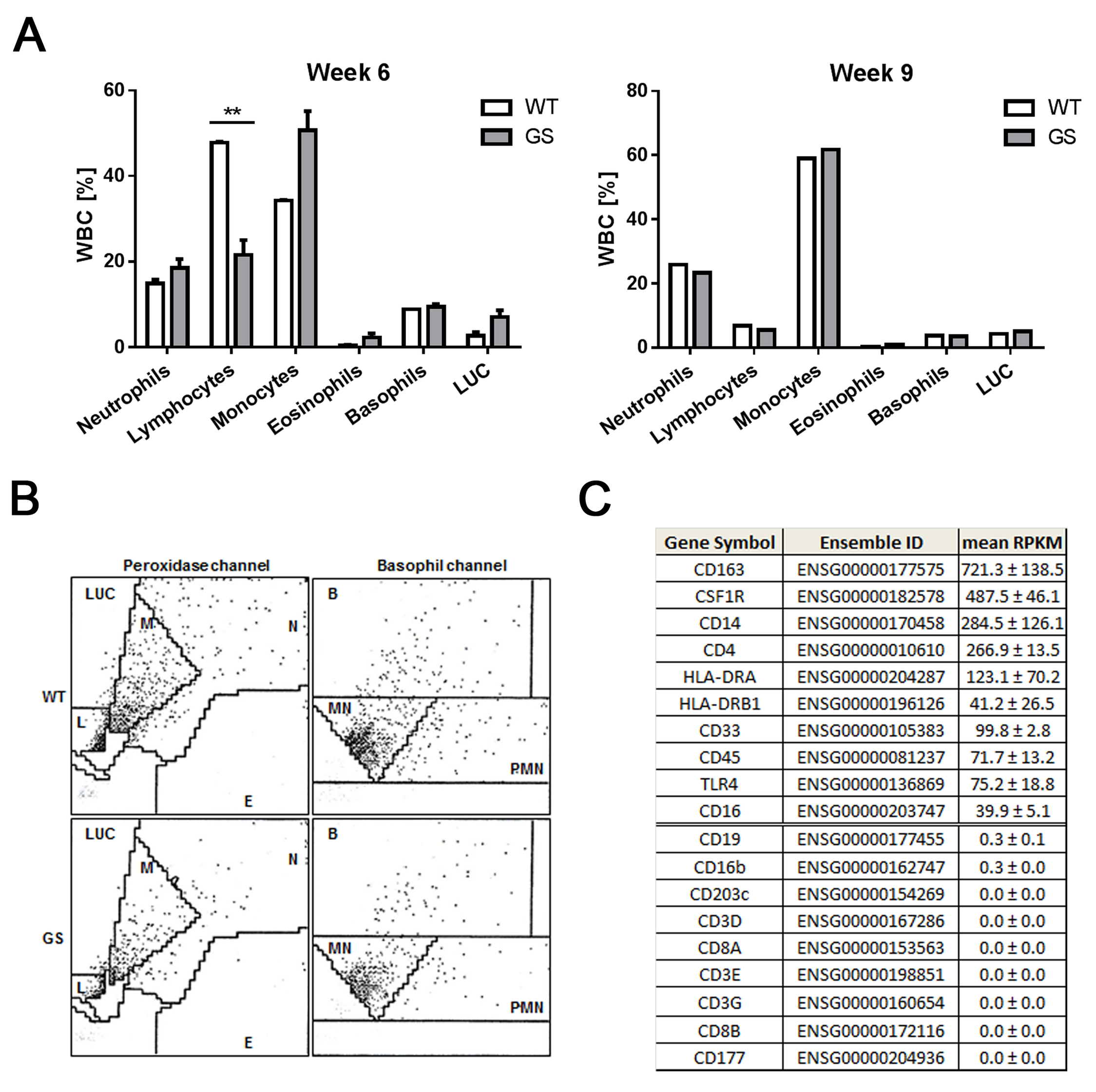

Supplement: S2 Fig — (A) ratios from white blood cell count (WBC) of the differentiated monocytes reveal a higher proportion of monocytes in LRRK2 (G2019S) (GS) patient cell cultures compared to gene-corrected wild-type (WT) control cultures after 6 weeks of differentiation (left panel; n = 3). After 9 weeks of differentiation, monocytes are the predominant cell type in cultures of both genotypes (right panel). Differences between genotypes are no longer observed. (B) Representative peroxidase (left column) and basophil (right column) cytograms obtained from leukocyte differential analysis of iPSC-derived monocytes. N: Neutrophils; L: Lymphocytes; M: Monocytes; E: Eosinophils; B: Basophils; LUC: large unstained cells; MN: mononuclear cells; PMN: polymorphonuclear cells. (C) Gene expression analysis of CD14+ FACS-sorted iPSC-derived monocytes after 16–19 weeks of differentiation. Genes with mean reads per kilo base per million (RPKM) > 5 were considered being expressed (n = 4; ±SEM). (TIF) [file pone.0165949.s002.tif]

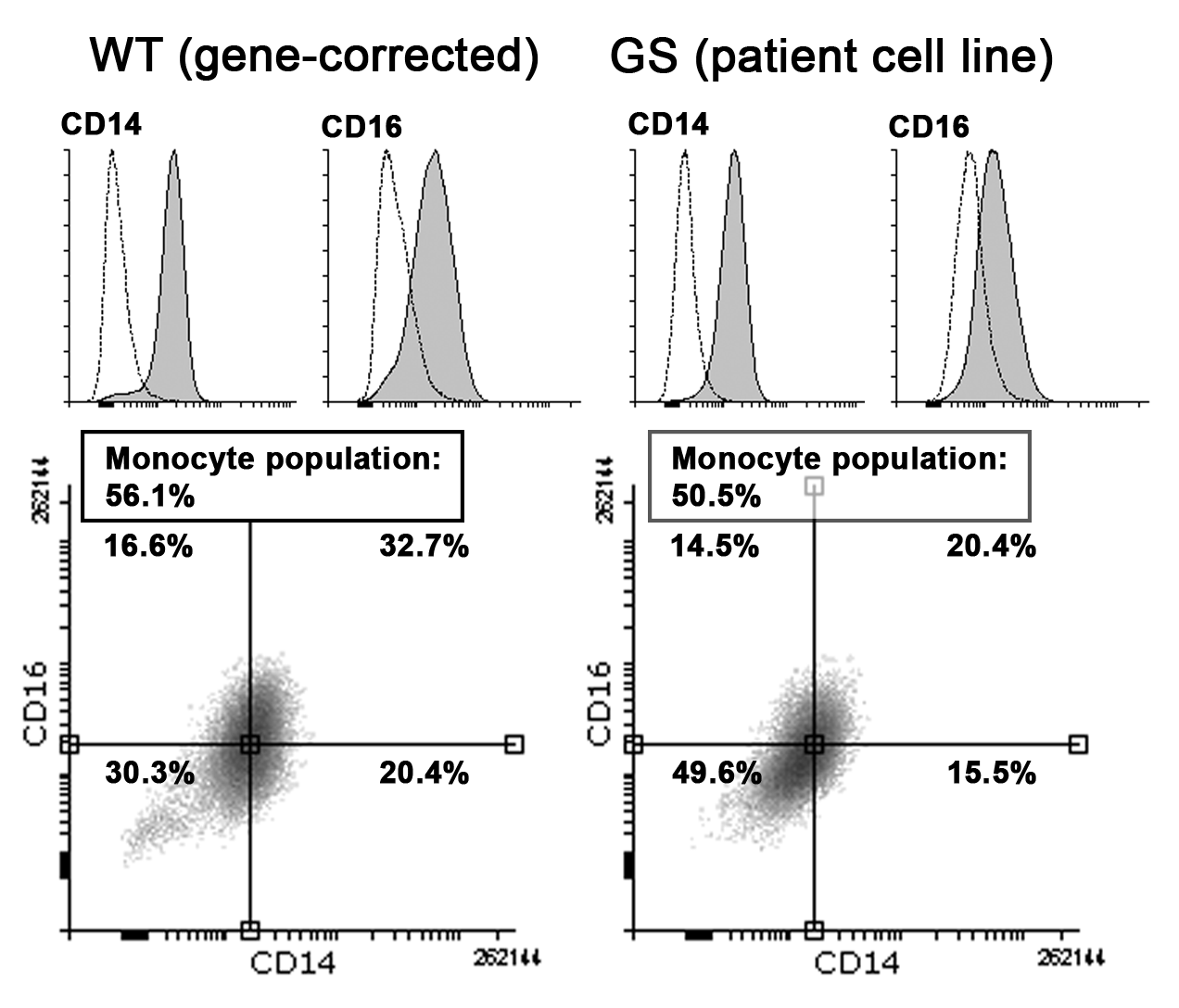

Supplement: S3 Fig — (A) Representative peaks of flow cytometric analysis of CD14 and CD16 in LRRK2 (G2019S) patient (GS) and gene-corrected control (WT) cells after 19 weeks of differentiation. Histograms represent specific surface staining (shaded grey) compared to unstained (dashed line) and isotype-matched (solid line) controls. Representative CD16 vs. CD14 scatter plots illustrate the distribution of the gated monocyte population, the respective monocyte yields (differentiation efficiency) are given in boxes. For each cell line, three 6-well plates, with each well containing 10–12 monocyte producing cell clusters, were set up for differentiation and analyzed in two experiments. (TIF) [file pone.0165949.s003.tif]

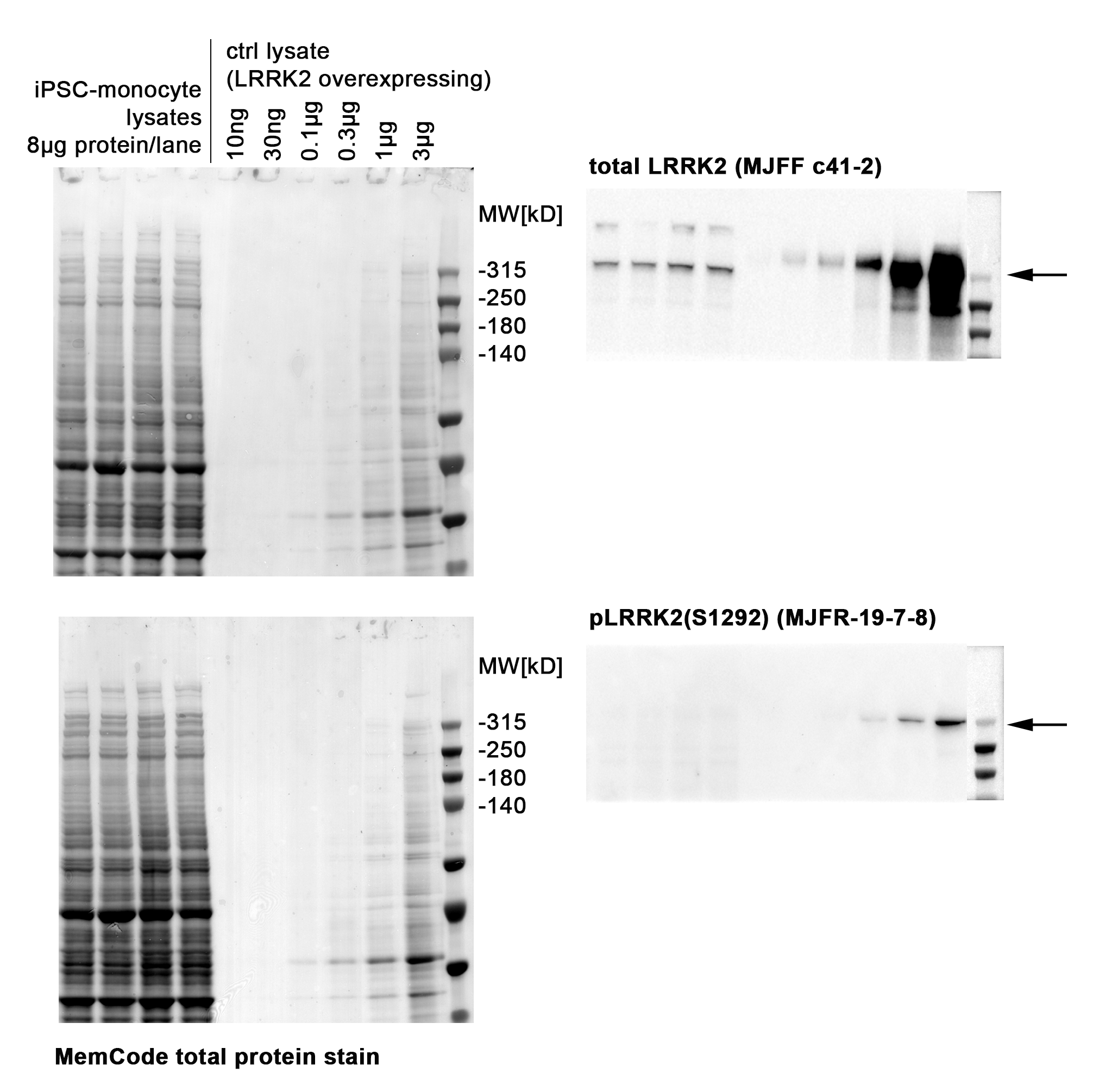

Supplement: S4 Fig — Representative immunoblots of iPSC-derived monocyte and tagged LRRK2 overexpressing cell lysates showing total LRRK2 (upper lane) and phospho-LRRK2(S1292) (lower lane) protein expression. Similar protein load was verified using Memcode total protein staining. Phospho-LRRK2(S1292) did not reveal any signal in iPSC-derived monocyte lysates. (TIF) [file pone.0165949.s004.tif]
